# The role of social support in the decision to migrate for childbirth: qualitative evidence from India

**DOI:** 10.21203/rs.3.rs-4839396/v1

**Published:** 2024-09-03

**Authors:** Rutuja Patil, Aanchal Narang, Alison M. El Ayadi, Kajal Tonde, Rachel Murro, Shivani Khadilkar, Dhiraj Agarwal, Sanjay Juvekar, Nadia G. Diamond-Smith

**Affiliations:** Vadu Rural Health Program, KEM Hospital Research Centre; Department of Epidemiology and Biostatistics, University of California San Francisco; Department of Epidemiology and Biostatistics, University of California San Francisco; Vadu Rural Health Program, KEM Hospital Research Centre; Department of Epidemiology and Biostatistics, University of California San Francisco; Vadu Rural Health Program, KEM Hospital Research Centre; Vadu Rural Health Program, KEM Hospital Research Centre; Vadu Rural Health Program, KEM Hospital Research Centre; Department of Epidemiology and Biostatistics, University of California San Francisco

## Abstract

Temporary Childbirth Migration (TCM) involves women returning to their natal homes during or after pregnancy, a common but understudied practice in India and South Asia. This study examines social support practices influencing TCM decisions among Indian women. Factors such as family support, especially from husband, in-laws and parents, play a crucial role in migration decisions during the perinatal period. Understanding these factors is essential for improving maternal and child health outcomes and for developing targeted policies and interventions. Cultural influences also contribute to TCM decisions, impacting the timing, duration, and motivations behind women’s choices to migrate during or after delivery.

Our study was conducted at the Vadu Health and Demographic Surveillance System (HDSS) in Western Maharashtra, India. We conducted 41 in-depth interviews with triads of women, their husbands, and mothers-in-law from Vadu HDSS, focusing on perceptions, timing, reasons, decision-making, and support related to Temporary Childbirth Migration (TCM). Participants varied in age, education, occupation, marriage type, migration type, delivery method, and included women who recently delivered or had infants up to two years old. The qualitative data were analyzed using both rapid analysis and traditional analysis using coded transcripts, incorporating both predefined and emergent codes to capture a wide range of participant characteristics and experiences. We later categorized our findings in Social support domains.

Social support, including emotional, financial, instrumental, and informational, is a critical factor in TCM. Emotional support from mothers is highly valued, providing a stress-free environment. Financial support from husbands, in-laws, or parents influences the decision, with financial responsibilities sometimes dictating the choice of residence. Instrumental support, such as help with household chores, is essential, with varying levels of support at different locations. Informational support from experienced family members also guides expectant mothers.

Healthcare access, household status, and the role of husbands further impact TCM decisions. Women prioritize proximity to medical facilities, comfort, and freedom in their natal homes. The inclusive decision-making process often involves women, in-laws, parents, and husbands. The study’s findings highlight the complex interplay of social support, cultural norms, and practical considerations in TCM decisions, underscoring the need for more research to understand and support women’s choices during the perinatal period.

In conclusion, social support is a key driver of TCM and women’s status in their households affects their support needs. Recognizing the importance of emotional, financial, informational, and instrumental support will help healthcare providers and policymakers to better assist expectant mothers, promoting positive maternal and child health outcomes.

## Introduction

Temporary Childbirth Migration is a practice common in much of South Asia, including India, in which a woman returns to her natal (her own parents) home at some stage during her perinatal period^1–3^. This pattern is driven by the underlying common practice of exogamy, where women marry someone often living in another village, district or state, and mostly live with their husband’s parents (her in-laws)^4^. Estimates from previous research by our team find that the prevalence of temporary childbirth migration ranges from around 30%-over 80%^3,5^.

While a new body of research is describing the patterns of temporary childbirth migration (duration, timing, etc.), little is known about factors driving this practice. Quantitative data from two states of India (Bihar and Madhya Pradesh) found that some women are more likely to return to their natal homes than others, specifically women are more likely to return for the first birth, and more educated, wealthier women are more likely to return.^1^ The most common reason that women reported returning to their natal home (reported by over 50% of women in both states) was that they will get better care, rest, or comfort at their natal home. A qualitative study in Mumbai also found that women wanted to migrate to receive more care and support, and because they expected to work less and have lower health-related costs^6^. Another study in Bangalore found that women highly valued the support of their own mothers during pregnancy, childbirth and postpartum, and mothers were seen as the most important support source during this time^7^. A qualitative study in Tamil Nadu found that returning to the natal family was critical for because the woman’s parents were expected to cover childbirth costs in addition to caring for her.^8^ Other studies of traditional pregnancy and childbirth practices mention the return to the natal home as providing mothers with sufficient rest and a break from their routine household chores, important for preventing infection, or influenced by views about ‘ritual pollution’ stemming from menstruation^9–13^. These suggest that there are various support roles that the natal family can play for women and that impact the decision to migrate.

Thus, while social support has been described as a factor influencing temporary childbirth migration, past research has not disentangled what types of “care” and “support” women want and feel that they do not get in the husband’s home. Understanding more about the types of care, or support, that women expect to get at their natal home, and how this plays into the migration decision-making process, will help us understand drivers of this phenomenon. Social support during the antenatal and postpartum period of pregnancy has been associated with higher levels of purported well-being and lower levels of depression among new mothers^14^; moreover, studies across the USA, Japan, Brazil, and India have shown that fewer available supportive persons during pregnancy is a predictive factor for antenatal and postpartum depression^15^, with women who have lower levels of social support having higher odds of developing suicidal ideation^16^. Most research on social support and pregnancy has focused on its effect on mental health, with less research looking more broadly at social support itself, which social support domains women value most, and how the desire for specific domains of social support being provided by specific support persons determines behaviors such as temporary childbirth migration.

Past theory has delineated four common types of social support: instrumental, emotional^17^, informational, and appraisal. There is some limited past research in South Asia has heighted the importance and different types of social support that women want during pregnancy and postpartum. In a study in Pakistan, pregnant women most commonly mentioned four types of social support that they highly valued: practical help with routine activities, information/advice, emotional support and assurance, as well as the provision of resources and material goods^18^. However, different people were seen to be best able or positioned to provide certain types of support, with mothers and mothers-in-law equally being mentioned for practice help with routine activities.

The majority of research on support in perinatal period has focused on women’s support at the time of delivery. A qualitative study in India highlighted the importance to women of having a support person at the time of delivery on their experience of the birth^19^. However, quantitative data from India found that 13% of postpartum women reported that they had no one to provide support in labor and 10% had no one to provide encouragement in labor (another indicator of support). Other indicators of social support were also low (helping them talk to providers, bring them food, etc.)^20^. Again, different people provided different types of support, with mothers/mothers-in-law providing encouragement (emotional) support and husbands providing instrumental (bringing food/water) support, for example. A systematic review of interventions (mostly in high income countries) to increase support for women at delivery (with a birth companion, doula, etc.) found increases in spontaneous vaginal birth, decreased use of anesthetics, higher APGAR score, and fewer negative feelings about childbirth^21^. A qualitative study about decision-making around where to deliver (private or public facility) in India found that perceptions about where women could get the most social support were important factors^22^.

While existing literature shows that supportive social factors during pregnancy, childbirth, and postpartum improves maternal experience and can potentially improve maternal health outcomes, there is little data on the impact of social support on the decision to migrate in the perinatal period. If women are seeking specific forms of support, and they perceive that their own mother, compared to their husband or mother-in-law, can provide that better, they may be more likely to choose to migrate in pregnancy, for example. The aim of this paper is to describe perinatal social support needs and preferences and explore how social support considerations influenced temporary childbirth behavior, and how social support norms and structure (health systems access) intersect with the desire for social support. Additionally, this paper considers the differing roles of women, their husbands, and their mothers-in-law on the decision to migrate temporarily or not. Finally, this paper considers the role of cultural factors and community norms surrounding Temporary Childbirth Migration.

## Methods

This study was conducted at the Vadu Health and Demographic Surveillance System (HDSS) in Western Maharashtra, India which longitudinally updates the demographic data and vital events of a population of 180,000 since 2002. Although the overall study is a mixed methods study, we have only reported qualitative findings in this paper. The study was approved by the KEM Hospital Research Centre institutional Ethics committee (KEMHRC/RVM/EC-1899 dated 29th September 2022) and the University of California, San Francisco IRB (UCSF IRB no 22–36484). The study was conducted as per the Indian Council of Medical Research- National ethical guidelines for biomedical and health research involving human participants. The data were collected after administering written informed consent of the participants.

We conducted a total of 41 in-depth qualitative interviews with triads consisting of the index woman, her husband, and her mother-in-law. The interviews were conducted by co-authors KT, RP and SK. Participants were selected from the Vadu HDSS data, ensuring maximum variation in demographic characteristics such as age (21–38), education level (no formal education-postgraduate), occupation (working/ non-working), marriage type (arranged or love), migration type and distance, as well as variables related to type of delivery (vaginal/ caesarean). Inclusion criteria comprised women who recently delivered or had infants up to two years old, while exclusion criteria included pregnant women. We collected data using the indepth interview guides which included sections on perceptions about TCM, timing and duration of TCM, reasons for TCM, decision making, factors affecting the decision, people involved, factors influencing the TCM, cultural practices or beliefs for TCM, impact on access to health etc. We also discussed the support (informational, emotional, esteem, and tangible) received by the participants which may have influenced the TCM and its decision.

We used two distinct methods of analysis. Initially, a rapid analysis method was used to assess and interpret iterative findings, and to guide ongoing in-depth interviews. To ensure accurate analysis, the interviews were recorded and later transcribed. These transcripts were then translated and meticulously coded both deductively and inductively using the qualitative analysis software Dedoose. We utilized predefined codes for consistency but also incorporated additional codes as identified during the analysis process (inductive and deductive coding process). We were theoretically informed by the four domains of social support^17^. Each transcript was double-coded by two members of the team to ensure coding reliability and uniformity. Coding was done by a three-person team based in the US, including two individuals of South Asian descent. Further analysis was done by members of the US and India based teams, and throughout the process there was constant communication between teams to ensure that interpretation was informed by the local context. Despite this, the perspective of western-born team members may have influenced some interpretation. All members of the coding and analysis team were also cis-gender women, some of who had gone through childbearing, and thus, the interpretation of data was influenced by our lived experiences.

## Results

Participants described a range of types of social support they desired, the different persons they wanted that support from, and how that impacted their behaviors around migration. They also described how other factors influenced their decision to migrate in pregnancy, and intersected with their desire for social support.

### 1. Social Support

#### a. Emotional Support

Emotional support during pregnancy and childbirth is an important aspect of overall well-being. Participants emphasized the benefit of emotional backing from family members, particularly their mothers and/or the husbands. The study participants highly valued the presence of a supportive environment where they felt understood, cared for, and free from stress and judgement. One participant shared: “*When I’m at my mother’s place, I feel free. There’s no tension, I can do whatever I want, everything revolves around me*.” (Age- 21 pregnancy #-2, Edu: Gaduate)

While another participant shared how her mother would provide psychological support and she would not have to worry about people being irritated with her:

“*Yes, psychological support is absolutely required, rest of things are immaterial. No irritation in any case. Mother never gets irritated…You experience everything afresh during your pregnancy and therefore only your mother is required to be nearby for psychological support*.”

(Age-27, pregnancy #1, Edu: Graduate)

This reflects the nurturing atmosphere that pregnant women or new mothers often seek, where their emotional needs are prioritized. It also highlights the potential fear women have about how they are perceived (being irritating, potentially) in their husband’s home.

#### b. Financial Support

Participants also highlighted the role of financial assistance from their husbands, in-laws, or parents for expenses related to pregnancy and childbirth. The finances included expenses incurred during pregnancy (including medical check-ups, sonographies, other necessities, and later for the delivery itself). The ability to access adequate healthcare, purchase necessary supplies, and cover medical expenses contributes significantly to the overall well-being of expectant mothers. One participant shared the following about her cousin’s experience:

“*At that time, she told me that her in-laws were saying that if you go to your mom’s place, they will take care of the expenses. So, her husband also said that you go there for the delivery, and even she was taking treatment in a private hospital, but they* [cousin’s in-laws] *insisted she go to the government hospital for treatment, so she told me that she was going to her mother’s place for the delivery. And I was not sure if I would go or not. But afterwards I felt like going to mother’s place so I went*.”

(Age-24, pregnancy #1, Edu: Graduate)

In contrast, another participant’s husband said he chose to keep his wife at their home due to his belief that he should pay the delivery costs:

“*If she is my wife, then why should I ask her parents for money and medicine?…Caesarean costs around 70,000 to 80,000 rupees at least. If it* [child] *is mine, then it should be my responsibility. Why should I let them* [his in laws] *do it? Of course, she can go afterward for a month if she wants to go*.”

(Husband, Age-37, primary school education)

Financial support was thus an element of social support that was considered in the temporary childbirth migration decision-making process, but could act in both directions.

#### c. Instrumental Support

Practical assistance with daily chores and caregiving is essential for pregnant women, particularly during the later stages of pregnancy and the postpartum period. Participants recounted the support received from family members, especially their mothers and mothers-in-law, in managing household chores, cooking, and caring for other family members. If the doctor recommends bed rest and there’s someone available in the marital family to manage household chores, women opt not to travel to avoid discomfort. However, if there’s no assistance available, the family chooses for women to relocate to the maternal household, allowing her to rest fully. Intertwined with the desire for help with household tasks, was a fear of a negative reaction from mothers-in-laws if she was perceived as not working enough, or adding work to others (namely, the mother-in-law).

“*I wanted to go natal home because you get to rest in the natal home…In the In-law’s home you cannot get it. Mother-in-law will scream if you do not complete it, or she does housework and she gets overburdened with housework. We also feel bad so that’s why going to a natal home is better*.”

(Age-38, pregnancy #-3, higher secondary education)

One mother-in-law described how she specifically called her daughter-in-law back to her marital home (away from the natal home) because she needed more help in the house:

*R: We hurried* [to bring the daughter in law back to marital home] *because then I could go to the farm… we thought that if we brought her here, then she would be a company and a person is required to do housework because it is quite troublesome to cook after coming from the farm, isn’t it?*

(Mother-in-law-Age-59, illiterate)

Availability of better care and support was one of the most common reasons for migration to natal home. However, not all women saw there as being better instrumental care at their natal home. Some participants described how the care they received from family members, particularly their mothers-in-law, played a crucial role in ensuring a smooth and comfortable pregnancy experience. For example, a mother-in-law explained how, given her own memories of how hard it was being pregnant as a daughter-in-law herself in her youth, she wanted to try to help her own daughter-in-law.

“*Talking about workload…if my daughter in law is sleeping anytime, I am the one to do all the work…I can understand that she is about complete nine months of her pregnancy and she doesn’t feel like doing (work) now… I look after her just the way I do with my daughter…I do her things. She is daughter in law… previously during our times things used to be like no matter how much mother-in-law asked to bring or do, we were asked to do all the work right up to the time of delivery, but I do not like to do that to her. I do not want her to suffer the way I have suffered… She also has same feelings as I used to have then I should keep my things in my mind and try to understand her*.”

(Mother in Law, Age-45, Secondary education)

Thus, there was variations in the level of instrumental support provided at different locations.

#### d. Informational support

Access to reliable information and guidance from experienced family members is crucial for pregnant women and new mothers. Participants expressed a preference for seeking advice from older family members, particularly elder women, who have first-hand experience with pregnancy and childbirth. Their wisdom and insights provide reassurance and guidance to expectant mothers during this transformative period. A participating husband emphasized this point, stating, “…*the actual person who has gone through this procedure, that person can guide you more accurately*.” Another participant shared it was her mother who could provide this best to her:

“*Yes, as the due date approaches, there is a fear in the mind about what to do. Now, what will happen? What will happen next? It means, when I get some information from my mom, it is like this or it happens like this, you don’t have to worry about it. That little information shared with my mom gives us some insights, and if it seems appropriate, then we feel like we should go to our mom’s place for a while. We should get information from her about what happens to us, and we should share it with her. That’s why we go there*.”

(Age-24, Edu: Graduate, pregnancy #-1)

The availability of elder women who can provide guidance was a primary factor in the decision to migrate during childbirth, and this could act as either a push factor to the natal home or pull factor to stay at the husbands home depending on where there were perceived to be more elder women to provide guidance. For example, a participant’s husband believed his wife should not migrate due to the perceived lack of delivery information at her natal home:

“*Because their side of people had no idea about the delivery, about anything. Her mother does the job and no one is there at home. Room is also smaller… They live there on rent, that’s the situation. And as my sister’s delivery was done by my mother only, she had the information. So that’s why*, [my mother] *said, let’s do it here only. My mother has all the information, so let’s do it here*”

(Husband- Age-29, Graduate with vocational diploma)

## 2. Other Factors Influencing temporary childbirth migration

### a. Access to health care facilities

Access to adequate healthcare facilities was also cited as an important consideration in determining the most suitable environment to be in during pregnancy, childbirth and into the postpartum period. A participant explained,

“*Although I wanted to stay with my own family, we decided to stay with my in-laws due to their support and the convenience of being closer to medical facilities*.”

(38, Higher secondary school, pregnancy # 2)

Women who had higher risk pregnancies were more likely to make decisions based on access to support and medical facilities. As one husband described:

“*The doctor told us that the baby is not growing well from 6th month of pregnancy due to high blood pressure. Due to this, we took the decision of migrating to maternal home even before dohale jevan [traditional baby shower.]*”

(Husband, 24, Graduate)

### b. Household status

Comfort during pregnancy and childbirth is paramount for expectant mothers, as it directly impacts their physical and emotional well-being. Participants described how them being able to meet their preferences surrounding eating practices and clothing contributed to their comfort during pregnancy. The ability to choose comfortable attire and adhere to dietary preferences reduced discomfort and enhanced overall satisfaction with the pregnancy and postpartum experience. This choice was often easier to exercise at maternal homes as compared to marital homes, where women have less decision making power. Relatedly, women’s social standing in the husband’s house, where she generally has low status, contributed to her overall feelings of power and freedom, and subsequent comfort and happiness.

*There were many such restrictions. It felt like being tied up in such bonds…our house doesn’t have the freedom to go out and roam freely like others. We need that freedom, right? To do small things or to organize something special for the children, we eagerly do things or celebrations for small children, right? Our (maternal) house gives a lot of freedom, unlike here. Only the husband supports me, but he is also answerable to his mother, so I feel suppressed because of that*.”

(Age-29, education B.A, pregnancy #-1)

### c. Role of Husbands

In cases where women decided not to migrate, they seek emotional support with their husbands instead of their mothers-in-law. Nonetheless, husbands may lack the knowledge and experience required to provide effective support during childbirth, and they may express apprehension about potential complications. The husband of a woman who has migrated during childbirth summed up the situation by saying:

“*They share it with the mother (pregnant woman’s mother). The mother has an experience. If she had stayed here, she could share things with me, I could possibly get happiness but there are few conditions in which I have no experience of, so I can’t tell anything. So the actual person who has gone through this procedure, that person can guide you more accurately. Like, “Dear, some distress is normal.” There are types of false labors 10 to 12 days earlier. Such things a mother would know better*.”

(Husband, Age 27, education BCA)

## Discussion

While the tradition of temporarily relocating to one’s natal home during the first childbirth is a prevalent practice, it is important to note that this decision is influenced by various factors. These factors encompass considerations such as the availability and competence of caregivers, access to transportation, and proximity to healthcare facilities in case of emergencies. Financial considerations such as the availability of resources and financial stability influencing the choice of where to stay during pregnancy and childbirth. Location choice also hinges on the availability of transportation and proximity to healthcare facilities, particularly in high-risk pregnancies or when the expectant mother faces potential health complications. The decision is further influenced by where the woman believes she will receive better post-delivery care. Aside from these more practical financial, informational, and instrumental forms of support, the desire for emotional support from maternal figures was cited as the most important consideration in determining the most suitable environment for pregnancy and childbirth. This is in line with previous research that highlighted the important role of emotional support from women’s mothers and their broader natal family in the perinatal period, and the vital impact that can have on mental health^14^. Our findings in reinforce the existing conceptualization of childbirth return as an act of “mothering the mother”, providing necessary support during a particularly vulnerable time period^23^. While prior studies have mentioned other forms of support (aside from social/emotional) including instrumental (for example woman’s parents covering childbirth costs) or tangible (postpartum women’s own mothers taking on household chores responsibilities), none of these prior studies looked across the different domains of social support. Our findings suggest that there are several different types of support roles that the natal family can uniquely play for women and that impacts why women want to return to their parents’ home.

Importantly, these factors can act as both push and pull factors–where different forms of support are stronger in the natal home, women may prefer going there, but in some cases women and their families perceive better care and support (of various forms) in the husband’s home. Many women express a preference for returning to their natal home during childbirth because they find reassurance and emotional support from their mothers, with whom they can confide. Newly married women may not have formed a close bond with their mothers-in-law since they are newly married, which adds to their inclination to choose their natal home. In other words, where relationships or resources in the husband’s home were stronger, women might stay, but where bonds and/or access to resources was stronger at the natal home, she might prefer to return. More research on what factors (type of marriage (love/arranged), actual socio-economic status of the respective households, etc.) could help us understand where women are most likely to get the care they need, and how to help women meet those goals.

Of note, for the most part the decision-making process was described as being inclusive, with women having a voice, and often both in-laws, parents, and husbands being involved in the process, which has been found in other studies in urban slum areas of the same state of India^24^. Given that this is a more rural area, this was somewhat surprising given low decision-making power that women often have in the India context around other types of household decisions^25^. However, prior research has found that women often do have more of a voice in decisions around childbirth than in other aspects of their lives^26^. Relatedly, women’s role in her household (her status or empowerment) also played a key role in her desires to migrate or not. Past research has found that women have lower status in their husband’s home compared to their natal home^27^, and thus, when women have the opportunity to return to their natal home, where they are more able to eat the food they want and express themselves as needed, especially during an emotionally and physically important and potentially challenging time, they may take advantage of that opportunity. Temporary childbirth migration may be a social sanctioned time for women to return back to a safer and more comfortable setting.

While this study has many strengths and adds to the literature by including multiple perspectives (women, husbands, in-laws) and diving deeply into an understudied topic, it also has limitations. Data was collected from only one district in one state of India, and thus, is not representative or generalizable to other parts of India or other countries. We know that prevalence of temporary childbirth migration practices differs across India, and it is very high in this part of India (~ 80%), hence why women migrate may differ here compared to places where its less common. Finally, data was collected in 2023 about women who delivered in the past year, which overlapped with significant COVID-19 waves in India. This may have impacted some women’s experiences of temporary childbirth migration during that time period, however, relatively few women mentioned this as something impacting their temporary childbirth migration behaviors.

To conclude, one of the main drivers of temporary childbirth migration is social support, but there are many forms of social support that women desire, and in some cases, these are better met at the natal, and in other cases the husband’s, home. Women’s status in their household, and the difference in the status between the two households, underlies and impacts her need for different types of support. By recognizing the importance of emotional, financial, informational, and instrumental support, as well as the complex factors influencing decision-making processes, healthcare providers and policymakers can better support expectant mothers and promote positive maternal and child health outcomes. Additionally, prioritizing comfort and accessibility of care ensures that expectant mothers receive the support and resources they need to navigate pregnancy and childbirth safely and comfortably.

## Data Availability

The coded data relevant to the paper will be uploaded on “https://repository.kemhrcvadu.org/index.php/home” at the time of publication of this paper.
